# Accelerated mass loss of Himalayan glaciers since the Little Ice Age

**DOI:** 10.1038/s41598-021-03805-8

**Published:** 2021-12-20

**Authors:** Ethan Lee, Jonathan L. Carrivick, Duncan J. Quincey, Simon J. Cook, William H. M. James, Lee E. Brown

**Affiliations:** 1 School of Geography and water@Leeds, University of Leeds, Leeds, UK; 2 School of Geography, Politics and Sociology, Newcastle University, Newcastle, UK; 3 Geography and Environmental Science, University of Dundee, Dundee, UK; 4 UNESCO Centre for Water Law, Policy and Science, University of Dundee, Dundee, UK

**Keywords:** Environmental sciences, Climate change, Hydrology

## Abstract

Himalayan glaciers are undergoing rapid mass loss but rates of contemporary change lack long-term (centennial-scale) context. Here, we reconstruct the extent and surfaces of 14,798 Himalayan glaciers during the Little Ice Age (LIA), 400 to 700 years ago. We show that they have lost at least 40 % of their LIA area and between 390 and 586 km^3^ of ice; 0.92 to 1.38 mm Sea Level Equivalent. The long-term rate of ice mass loss since the LIA has been between − 0.011 and − 0.020 m w.e./year, which is an order of magnitude lower than contemporary rates reported in the literature. Rates of mass loss depend on monsoon influence and orographic effects, with the fastest losses measured in East Nepal and in Bhutan north of the main divide. Locally, rates of loss were enhanced with the presence of surface debris cover (by 2 times vs clean-ice) and/or a proglacial lake (by 2.5 times vs land-terminating). The ten-fold acceleration in ice loss we have observed across the Himalaya far exceeds any centennial-scale rates of change that have been recorded elsewhere in the world.

## Introduction

Meltwater released by Himalayan glaciers forms the headwaters of the major river systems in Asia, supporting food and energy production downstream, as well as maintaining a range of ecosystems and ecosystem services^1–3^. Ongoing recession and thinning of Himalayan glaciers raises concerns about the sustainability of water supply in the region^1,3–6^. Recent rates of ice loss across the region exhibit marked spatial and temporal variability^4,5^ but there is consensus that recession has accelerated over recent decades^7,8^. However, there has been no consideration of whether the magnitude and rates of glacier mass loss presently reported across the Himalaya are unusual in a longer-term (centennial-scale) context.

Long-term fluctuations of mountain glaciers are known to be controlled primarily by regional climate and topography^9,10^. Variations in the South Asian monsoon affect the southerly High Mountain Asia (HMA) mountain ranges and the mid-latitude westerlies are particularly relevant to understanding the glaciology of the western mountain ranges^11,12^ and both these two atmospheric systems interact with the Tibetan anticyclone, but the exact relationships that describe glacier response to changes in precipitation and temperature are poorly constrained^13^. Local factors such as the presence or absence of a debris layer or proglacial lake can play an important role^14–16^, although the overall impact of surface debris on ablation remains unclear^17,18^. Internally-consistent methods that robustly quantify changes exhibited by hundreds to thousands of glaciers at a time are required if such knowledge gaps are to be filled^7,8,19^.

**Figure 1:**
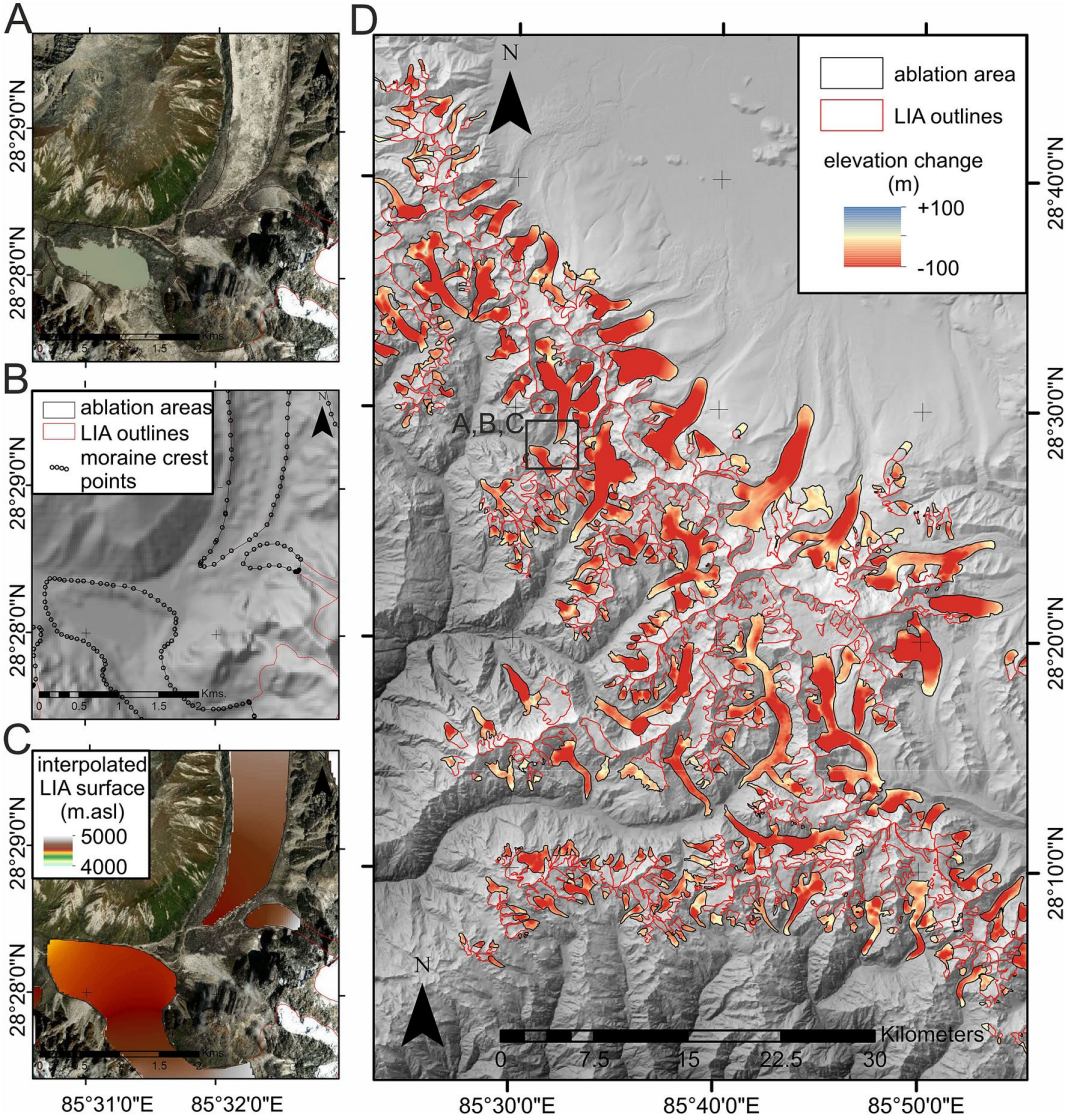
Example from the Langtang region of the Himalaya, illustrating geomorphological evidence comprising moraines and trimlines (**A**) used to delineate past glacier extent (**B**) and to reconstruct former glacier surfaces (**C**). Differencing of the reconstructed surface with a contemporary digital elevation model was used to quantify elevation change (**D**). The dataset analysis and preparation of this figure was made using ESRI ArcGIS software (v. 10.6).

In this study, we made the first assessment of Himalayan glacier changes over a centennial time scale. Specifically, this assessment was achieved using a combination of geomorphological mapping and palaeo-ice surface reconstruction techniques. We inferred the former extent (Fig. 1A,B) and surface elevation (Fig. 1C) of 14,798 glaciers during the Little Ice Age (LIA) maximum. The LIA was a period of pronounced climate cooling that culminated between 400 and 700 years ago across the Himalaya^13^. It represents the last period of widespread glacier expansion in the Himalaya and is therefore the benchmark position from which modern glaciers are currently receding. Our regional-scale analysis spanned ~ 2300 km of the Himalaya, across 20 degrees of longitude from Spiti Lahaul, India in the west, to Bhutan in the east and is unprecedented in its spatio-temporal coverage and density. Mapped glacier extents were used to quantify spatially-distributed elevation change (Fig. 1D) and hence glacier-specific volume change and mass balance from the LIA to present.

### Glacier area, volume and mass changes

**Figure 2:**
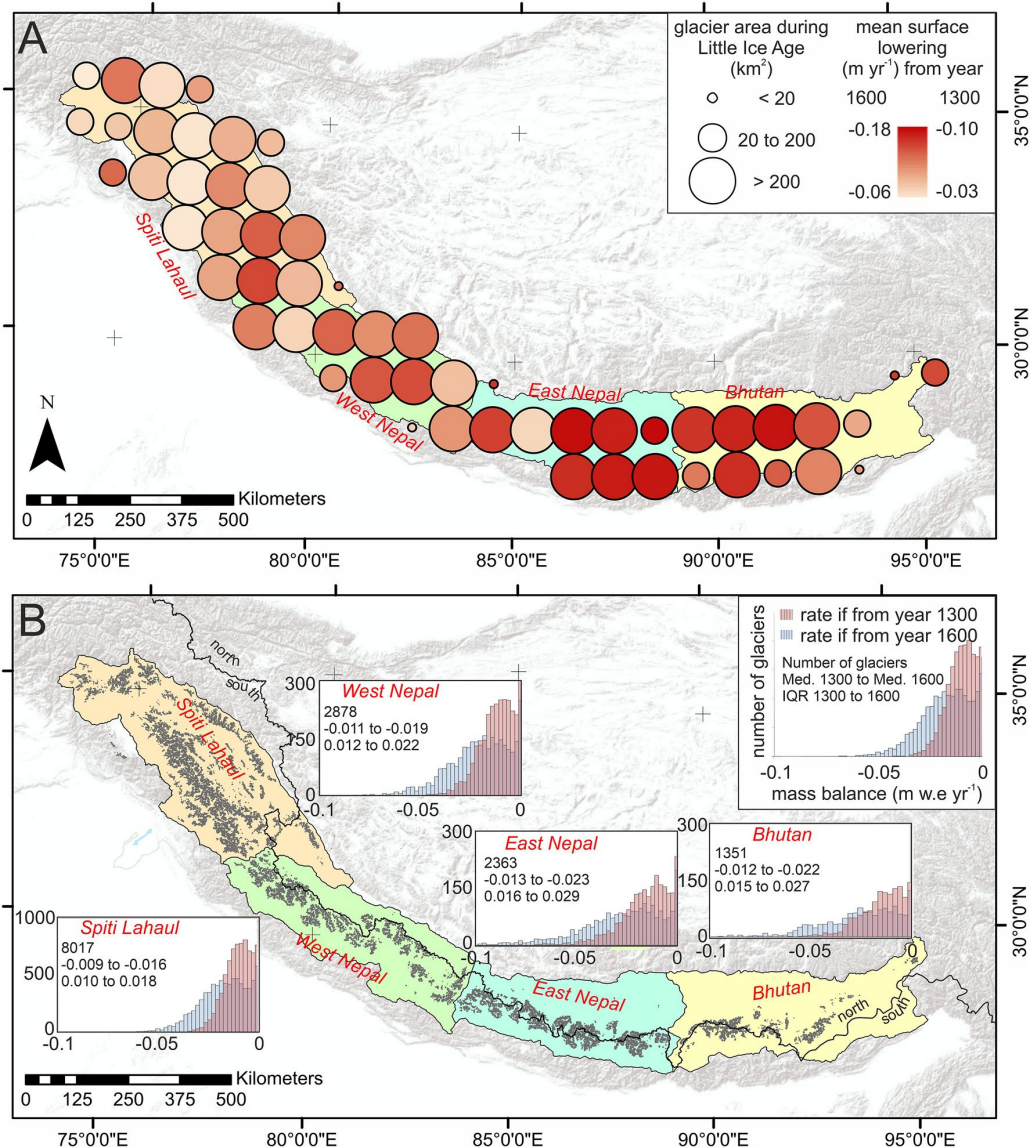
Surface lowering binned per degree of latitude/longitude (**A**) and mass balance (**B**) of glaciers across the Himalaya since the Little Ice Age. The thick black line in panel B denotes the north–south topographic divide. Note the larger y-axis range in panel B for Spiti Lahaul. The dataset analysis and preparation of this figure was made using ESRI ArcGIS software (v. 10.6).

Himalayan glaciers (n = 14,798) covered ~ 28,000 km^2^ at the LIA maximum. The contemporary area of these glaciers (n = 19,484) according to the Randolph Glacier Inventory^20^ is ~ 19,600 km^2^. LIA glacier coverage across the Himalaya was therefore at least 40% greater than at present. This is a conservative area loss estimate given our mapping protocol; see Supplementary Information (SI). Areal losses since the LIA were proportionally consistent between the major regions of Spiti Lahaul, West Nepal, East Nepal and Bhutan (Fig. 2).

Total Himalayan glacier volume loss since the LIA maximum was between 390 km^3^ and 586 km^3^. Relatively small losses can be seen around the terminus areas of most glaciers, with maximum surface lowering occurring several kilometers up-glacier, particularly on large valley-floor glacier tongues (1.7% of total glacier area) where lowering commonly exceeds 100 m (Fig. 1D). Rates of surface lowering ranged from − 0.03 m/year to − 0.18 m/year (Fig. 2A) and the greatest rates of change were in the eastern Himalaya (Fig. 2A).

Regional contributions to absolute volume loss were disproportionate given that the total number and density of glaciers are both highly spatially variable. For example, at the LIA maximum, Spiti Lahaul had 55% of the total number of glaciers and 40% of the glacierised area but contributed only 30% to the total measured volume change across the broader region. In contrast, East Nepal, with just 16% of the total number of glaciers at the LIA maximum, and 25% of the glacierised area, contributed 35% of the total ice volume loss. In comparison, West Nepal and Bhutan had 20% and 9% of glaciers at the LIA, and contributed 22% and 13% of volume loss, respectively.

Estimates of long-term mass balance do not differ markedly between sub-regions, with East Nepal and Bhutan showing the most negative median rates overall, at − 0.013 to − 0.023 m w.e./year and − 0.012 to − 0.022 m w.e./year, respectively (Fig. 2B). The same two regions also showed the greatest within-region heterogeneity, with distinctly wider inter-quartile ranges in mass balance when compared to the two regions of Spiti Lahaul and West Nepal further west (Fig. 2B).

### Influence of debris cover and terminus type

To understand spatially-distributed differences in glacial mass balance rates since the LIA, glaciers were assigned to one of four mutually exclusive groups depending on their (contemporary) surface characteristics (debris-covered vs clean-ice) and their (contemporary) terminus environment (lake vs land). Combined groups were very heterogenous in number of glaciers: debris-lake (1%), debris-land (7%), clean-lake (4%) and clean-land (88%).

**Figure 3:**
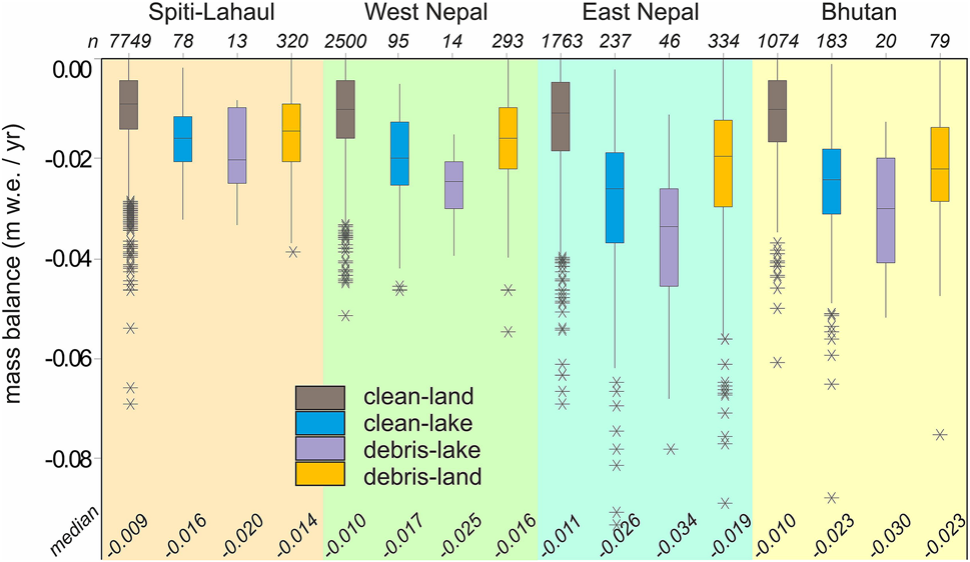
Influence of terminus type per sub-region (arranged west to east) on glacier mass balance. Inter-quartile range and outliers are indicated by boxes and asterisks, respectively.

Using generalised linear models (GLM), we identified significant differences in mass balance of all glaciers north versus all glaciers south of the main divide, between regions, and between glacier-terminus types (Fig. 3). See SI for summary statistics and median rates of mass loss for each group. The difference in glacier changes between north and south (Supplementary Fig. SI 2) most likely reflects large-scale atmosphere–topography interactions, which serve to bring orographically-enhanced precipitation to the southern slopes for example, as well as more local effects such as shading, particularly for those glaciers flowing to the north. East Nepal experienced the most negative mass balances for all terminus types (Fig. 3). In all regions, clean-lake, debris-land, and debris-lake glaciers had more negative mass balances compared to clean-land glaciers (Fig. 3; Supplementary Fig. SI 2). Clean-lake glaciers account for only 4% of the total number of glaciers in the Himalaya but contributed a disproportionately large amount to the total volume loss of around 14% (Supplementary Fig. SI 2).

Lake-terminating glaciers (clean-lake and debris-lake) had a median rate of mass loss that was two and a half times more negative (Fig. 3; *p*-value =  < 0.01; Supplementary Table SI2) than that of clean-land or debris-land glaciers. Examining these lake-terminating glaciers south and north of the main divide shows that they lost 40.6 km^3^ ice and 69.4 km^3^ ice, representing 13% and 40% of total ice loss on each side of the divide, respectively (Supplementary Fig. SI 2).

The presence/absence of a debris-cover exerts a strong control on rates of mass loss that is largely identical north and south of the topographic divide; debris-land and debris-lake glaciers combined contributed 47% (south) and 46% (north) to total volume loss (Supplementary Fig. SI 2), despite comprising only 6% and 9% of the number of glaciers, respectively. Separately, it is debris-covered glaciers without a lake that make the greatest contribution to mass loss because of their larger number, accounting for 38% of the loss overall. The median mass balance of all debris-land glaciers has been two times more negative than that of clean-land glaciers (Fig. 3; *p*-value =  < 0.01; Supplementary Table SI2).

Comparing the median rates of mass loss between debris-land and debris-lake we find that lakes have enhanced mass loss by − 0.01 m w.e./year. Similarly, comparing the median of the clean-lake group with that of the debris-lake group suggests an enhancement in mass loss by debris cover of − 0.07 m w.e./year. The additive effect of these two glaciological attributes becomes apparent when reminded that clean-ice glaciers had a median mass loss of 0.01 m w.e./year and that debris-covered glaciers that terminate in lakes have experienced a median rate of mass loss of − 0.027 m w.e./year, which is the most negative glacier-specific mass balances of any group of glaciers in all regions (Fig. 3).

### Controls on volume loss and mass balance

Our comprehensive dataset of LIA glacier extents allows us to make the first empirical investigation of the controls on long-term (centennial-scale) Himalayan glacier change. It is well known from quantification of recent (decadal) glacier changes during the satellite era that glaciers towards the east of the Himalaya are particularly sensitive to changes in air temperature^21^, amplified by variations in the seasonal precipitation regime^22,23^. These studies also show rates of mass loss to be largely uniform across Spiti Lahaul and East and West Nepal, with slightly elevated rates of mass loss recorded in Bhutan. The east–west pattern likely reflects spatially-variable temperature changes during the late twentieth/early twenty-first century^24^ and a recent weakening of the monsoon^25^. Our data, on the other hand, capture long-term and aggregated glacier changes, which are likely affected by changing temperature and precipitation patterns across different sub-regions and the subsequent timing of deglaciation^26^. The longitudinal gradient (*p* = 0.03; *r*^2^ = 0.06) in our results of both surface lowering and mass balance (Supplementary Table SI3), which shows a clear increase in mass loss in eastern regions compared to those in the west (Fig. 2), is much stronger than suggested by studies focusing on decadal-scale mass-balance and surface dynamical changes^5,27^.

Local-scale variations in terminus condition and surface cover are superimposed on these regional trends and play a crucial role in determining rates of change (Fig. 3). Debris-cover is a key factor in this local variability, with our results suggesting their long-term rate of mass loss outstrips that of their clean-ice counterparts by a factor of 3. This is perhaps surprising given that debris covers exceeding a critical thickness (~ 0.02 m) tend to reduce melt when compared to clean-ice glaciers at the same elevation^28,29^. However, several studies of contemporary mass loss have found comparable thinning rates over clean and debris-covered glaciers^4,30^. The physical processes underlying this anomaly are thought to include locally-enhanced ablation owing to ice cliff and pond development^18,31,32^ and decreased ice fluxes from the accumulation area, particularly where tongues have become dynamically detatched^33^. Over centennial timescales, a more plausible explanation is that these glaciers have been sustained at lower elevations than would otherwise be possible and therefore have large areas of ice available for ablation due to climatic forcing^14,34,35^.

Lakes at glacier termini exert a number of local thermo-mechanical effects causing ice surface drawdown and glacier acceleration, and hence enhanced ice mass loss^7,36,37^. Glaciers developing lakes at their termini therefore have an evolution of mass balance that is non-linear over decades to centuries^15,38^. Although glaciers with lakes are low in number (relative to those without) across the Himalaya, our results show, unequivocally, that they contribute a disproportionate amount to overall ice loss. It is likely therefore that lake development will exert an increasingly strong control on future Himalayan glacier mass balance as they grow in both number and volume^39^.

### Comparison with contemporary rates of change across the Himalaya

Centennial-scale rates of change provide a longer-term context to reported changes of Himalayan glaciers over the last few decades (Supplementary Table SI3). Crucially, our analysis reveals that glacier changes (median − 0.011 to − 0.020 m w.e./year) since the LIA maximum (1300 CE to 1600 CE, respectively) are an order of magnitude lower than reported for recent decades; for example Gardelle et al.^4^ for all Himalayan sub-regions (mean − 0.35 m w.e./year), Kääb et al.^40^ for 2003 to 2008 (mean − 0.42 m w.e./year), Brun et al.^5^ for 2000 to 2016 (mean − 0.36 m w.e./year), Maurer et al.^8^ for 1975 to 2000 (mean − 0.21 m w.e./year) and for 2000 to 2016 (mean − 0.43 m w.e./year) and Shean et al.^41^ for 2000 to 2018 (mean − 0.19 m w.e./year), albeit with some slight variations in the exact sub-regions studied. This comparison demonstrates that the mass balance of glaciers across the Himalaya has become dramatically more negative in recent decades in response to climatic forcing.

### Comparisons to other world regions

Studies from several other parts of the world have shown that recent decadal rates of glacier mass loss are greater than centennial-scale average losses since LIA, but not on the same scale as those detected for the Himalaya. For example, compared to the LIA-present average rate, there has been a doubling of mass loss for the Vatnajökull ice cap in Iceland between 2002 and 2010^42^, a 23% acceleration of mass losses in NE Greenland between 1980 and 2014^43^, a doubling of mass loss since 1986 for Patagonia^44,45^, and a doubling of losses for the Southern Alps of New Zealand between 2009 to 2019^46^. The order of magnitude increase in Himalayan glacier mass loss in recent decades compared to the post-LIA average therefore represents the most dramatic glacier response of any world region.

In conclusion, this study provides the first self-consistent measurement of Himalayan glacier extent and surface elevation during the LIA maximum across the entire Himalaya. The area loss of Himalayan glaciers since the LIA has been at least 40% of the LIA extent. Ice volume loss since the LIA has been between 390 and 586 km^3^, which equates to between 332 and 498 Gt mass equivalent and to between 0.92 and 1.38 mm Sea Level Equivalent (SLE). For the first time, we have identified that the centennial rate of mass loss since the LIA is an order of magnitude lower than the rates reported for the last decades. We suggest that nested scales of effects influence these rates of mass loss over a centennial time scale, namely (i) regional climate via the differences north versus south of the main divide and between morpho-climatic regions, and (ii) local topography and glacier morphology via terminus environment and debris-cover. The greatest rates of mass loss are in the eastern Himalaya. Whilst clean-land glaciers produced the most mass loss overall due to their number and size, glaciers with debris-cover had more negative mass balances and those with lakes the most negative mass balances.

Our study highlights three important points. Firstly, by comparison to other world regions the magnitude of the acceleration in glacier mass loss across the Himalayan region is exceptional. Secondly, whilst centennial-scale changes in climate forcings alter spatial gradients in regional glacier mass balance, it is local topography and glaciological attributes such as debris cover and the presence of a proglacial lake that promote rapid mass loss^7,14,47^. Thirdly, those attributes need to be accounted for in numerical models^15,38^ because they important drivers of mass loss. Overall, quantification of past glacier variability over a centennial timescale should help validate climate-glacier numerical models and thus produce more reliable projections of future mass loss under a warming climate^48^.

## Methods

### Little Ice Age moraine mapping

LIA glacier extents were mapped by extending the RGI 6.0 inventory outlines^20^ down-valley to the crests of moraines interpreted to represent the LIA maximum. Specifically, we delimited the innermost prominent moraine ridge down-valley of the contemporary glacier termini, where the ridge was often sharp-crested and with unstable, steep sides devoid of vegetation. These innermost ridges have in some valleys been dated to the LIA and contrast with other parts of multi-ridged moraine complexes that are more subdued, vegetated and more likely represent late Holocene ice advances^13^. Furthermore, choice of the innermost moraines maintains consistency, permitting replicability, and provides a minimum estimate of LIA glacier extent and hence a minimum LIA volume and mass.

### Ice surface reconstruction

For each of our LIA outlines, ablation zones were delineated by calculating areas below the equilibrium line altitude (ELA). Glacier specific ELAs were defined using the Area-Altitude Balance Ratio (AABR) method, automated using code developed by Pellitero et al.^49^. We specified a BR (balance ratio) of 1.75 as suggested by Rea^50^ for Himalayan glaciers. The area below each ELA was then extracted in an automated fashion, as described by Carrivick et al.^43^. The vertices of each ablation area were converted to points to enable extraction of elevations from a digital elevation model (DEM); i.e. of terminal and lateral moraine crests and of the ELA. A surface was interpolated between those points to represent the LIA glacier surface. Calculating the difference between our LIA surface and the contemporary DEM indicated the surface lowering that has occurred since the LIA to the date of the DEM. We then converted that to a glacier-specific volume change and a mean annual rate of change. The effect of DEM source (resolution or timing; Supplementary Fig. SI 1) on our calculations of volume was negligible; either using the ALOS 30 m DEM (www.eorc.jaxa.jp/)^51^ or the HMA 8 m DEM (www.nsidc.org)^52^. The effect of our choice of interpolation method on volume calculations was also small (Supplementary Table SI1).

### Rates of change and uncertainty

We report non-parametric statistics because our mass loss data are non-normally distributed, as they pertain only to glacier ablation areas. Rather than volume uncertainty, by far the biggest effect on our rates of change is the date ascribed to the LIA maximum. Rowan’s^13^ analysis shows that the LIA across the Himalaya was between 1300 and 1600 CE. Therefore, recognizing that not all Himalayan glaciers will have reached their maximum extent at the same time, we provide reasonable lower and upper bounds by computing rates of glacier change for both 400- and 700-year time periods. Between the time of the LIA maximum and the present, we acknowledge that rates of glacier mass loss may have varied. Episodes of stagnation and minor advances may have occurred, but these cannot be accounted for in this study. The SI details our consideration of the timing of the LIA maximum across the Himalaya, while also providing a description of our datasets, spatial analysis methods and our uncertainty assessments, and a summary of our statistical assessment of differences between groups (north/south of main divide, regions, terminus type; Supplementary Tables SI2 and SI 3).

## Supplementary Information

Supplementary Information 1.

Supplementary Information 2.

Supplementary Information 3.

## Data Availability

Our study utilized publically available datasets as cited and referenced. Our study produced new datasets; glacier outlines for the LIA and an ice surface reconstruction for the LIA, which are available as shapefiles and geotiffs, respectively, from https://doi.org/10.5518/939.
