# Supplementary material for: Accelerated mass loss of Himalayan glaciers since the Little Ice Age
Source: Sci Rep. 2021 Dec 20;11:24284. doi: 10.1038/s41598-021-03805-8 (PMC8688493; doi:10.1038/s41598-021-03805-8)
Supplement: Supplementary file 1 — Supplementary Information 1. [file 41598_2021_3805_MOESM1_ESM.docx]

Supporting Information for

**Accelerated mass loss of Himalayan glaciers**

**since the Little Ice Age**

Ethan Lee^1,2^, Jonathan L. Carrivick^1*^, Duncan J. Quincey^1^,

Simon J. Cook^3, 4^, William H.M. James^1^, Lee E. Brown^1^

^1^ School of Geography and water@leeds, University of Leeds, Leeds, UK

^2^ School of Geography, Politics and Sociology, Newcastle University, Newcastle, UK

^3^ Geography and Environmental Science, University of Dundee, Dundee, UK

^4^ UNESCO Centre for Water Law, Policy and Science, University of Dundee, Dundee, UK

**Contents of this file**

- Text on LIA dates of moraines
- Text on glacier geometry reconstruction
- Text on spatially distribution variability in glacial mass loss
- Figures SI 1 and SI2
- Tables SI 1, SI2 and SI3

**Additional Supporting Information**

available from <https://doi.org/10.5518/939>

- **Data Set S1.** LIA glacier outlines and ablation areas as shapefiles in UTM zone 45N projection.
- **Data Set S2.** LIA glacier ablation area surface elevation and lowering maps as geotiffs in UTM zone 45N projection.

**LIA dates of glacier moraines in the Himalaya**

Rowan (2017)^1^ reviewed and analysed available moraine ages attributed to the late Holocene and Little Ice Age (LIA) across the Himalaya. In that study, statistical analysis of 66 ages defined three distinct periods of glacial advance at ~400 AD, ~800 to 900 AD, and ~1300 to 1600 AD. These times of glacial growth match well with European LIA glaciation^2^. The last period of advance, 1300 to 1600 AD, was identified by Rowan^1^ to be the most widespread and thus was determined as the LIA maximum within the Himalaya.

The exact timing of maximum advance during the LIA will have been different for each glacier, and overall asynchronous across the Himalayan region. However, Rowan^1^ noted that the spatial distribution of dated samples also varies, with the majority taken from within the Central Himalaya region. Only 8 glaciers outside Central Himalaya have dated moraines to within the LIA. From an analysis of their controlling factors, neither altitude nor longitude affected the LIA moraine ages, while an influence from latitude was identified, with ages occurring earlier at lower latitude. However, due to the spatial clustering of the dated samples, any inferences of a spatial pattern for the whole Himalaya is impossible to determine^1^. The few more recent studies^3, 4^ of individual glaciers or glacierised valleys and are not enough to enable any determination of spatial patterns in the timing of the LIA across the Himalaya. Therefore, in this study we calculated our rates of glacier change for the upper and lower bounds of this time range; specifically, a faster rate from 1600 to present, and a slower rate from 1300 to present.

**LIA glacier ablation area surface reconstruction**

***Spatial analysis method***

1. In ArcMap (v. 10.4.1), and using spatial analyst extension, we mapped LIA limits of glaciers reported in the literature with LIA dates.
2. Manually digitised contiguous moraine crests and trimlines using 8 m HMA DEM and very high resolution (< 1 m) satellite imagery and aerial photography available in ArcGIS (ESRI software) basemaps. We used the RGI outlines of contemporary glaciers as a starting point and edited them to LIA limits.
3. Note that for some modern glaciers LIA moraine evidence was absent or obscured (by snow cover or cloud cover and/or poor quality DEM), and note that many of these LIA glaciers are a coalescing of several modern glaciers. Then the final number of LIA glaciers identified in this study n => 14,000.
4. Use Pellitero et al.^5^ (python) code to derive an ELA for every glacier by specifying a BR (balance ratio) of 1.75 as suggested by Rea^6^ for Himalayan glaciers, and then our own extension to that code (combined into a new stand-alone ArcGIS tool) to use the ELA to derive an ablation area automatically for every LIA glacier outline by intersecting each ELA contour with each glacier outline.
5. To use the ablation area polygons with the modern DEM to reconstruct a LIA ice surface for each ablation area, first ‘Densify’ ablation area outlines to add vertices evenly along all polygon edges (or else the parts that we digitised versus the parts from RGI 6.0 would be very different).
6. ‘Simplify polygon’ to remove extraneous LIA ablation area polygon vertices [and thus speed up subsequent computations].
7. Convert the ablation area outline ‘feature vertices to points’. i.e. to extract the points from the ablation area shapefile.
8. Use the extracted ablation areas to ‘extract by mask’ the contemporary DEM.Use these points to ‘Extract values to point’ from DEM; i.e. to assign elevations to the moraine crests and to the rest of the ablation area outline.
9. Interpolate a LIA glacier surface between these aablation area elevation points using extracted rastervalue from step 8
10. Determine surface elevation change between the LIA and present by computing LIA surface minus DEM = surface lowering.
11. Use zonal statistics to determine sum elevation change for each glacier ablation area and multiply by cell size to get volume change per glacier.

***Data sources, spatial analysis method and uncertainty in estimates***

Rowan^1^ summarised that across the Himalaya two distinct sets of moraines are commonly found in front of modern glaciers. The inner moraines (determined to be LIA in origin) are distinct morphologically from the outer (older, late Holocene) moraines, with steeper-sided ridges and ‘fresher’ (appear lighter coloured due to being unstable and largely devoid of vegetation) surfaces compared to more subdued and multiple ridge complexes of the former. LIA moraines were identified to be of most prominence close to the current glacier terminus, thus any subdued moraine behind these were determined to be recessional and older in nature and origin.

We did not map LIA outlines where geomorphological evidence was absent or ambiguous, where snow cover or cloud cover obscured the ground, or for small and steep hanging glaciers (permanent ice at altitude that is not situated in a well-defined cirque or depression).

We always digitised the innermost prominent (assumed to be LIA) moraine crest for consistency and for provision of a conservative estimate of ice extent and volume during the LIA. We cannot account for ice that occupied local topographic basins now filled with water but we expect this volume is negligible to the total. Our LIA glacier reconstruction technique pertains only to glaciers where the Randolph Glacier Inventory (RGI 6.0)^7^ outlines exist and we note there are many empty cirques that might have held small glaciers during the LIA; this ice contribution is not included in LIA-to-present volume change estimates. Nonetheless, we certainly account for the vast majority ice volume lost since the LIA because it will have been located below the LIA equilibrium line altitude (ELA) which we have incorporated through our derivation of glacier-specific LIA ablation areas.

We calculated the change in glacier volume from the LIA to the present day by differencing a LIA glacier ablation area surface with the modern DEM. Following the method of Carrivick et al.^8, 9^, LIA glacier ablation areas were produced by automatically estimating glacier-specific ELA using the Area-Altitude Balance Ratio (AABR) method and the ArcGIS tool developed by Pellitero et al.^5^. These polygons were subsequently merged with our mapped moraine crests and trimlines. The modelled glacier-specific ELAs fitted the geomorphological evidence, i.e. the maximum height of lateral moraines^10^ well. Surface lowering was converted to a volume change estimate by summing the grid cell elevation changes for each glacier zone and multiplying by cell size. This method excludes any thinning and hence volume change that might have occurred above the LIA ELA, i.e. in glacier accumulation areas, but we do not account for this because (i) there is no geomorphological evidence of it, (ii) DEM data are more likely to be missing or of poor quality is within glacier accumulation areas, and we re-iterate therefore that our estimates of volume loss since the LIA to the present are conservative.

Where our workflow identified positive elevation changes (Fig. SI 1A) i.e. a present day surface higher than the reconstructed LIA surface, we examined these manually on a case-by-case basis. Some were due to surges and are presumably cancelled out in the volume change estimates by a corresponding area of elevation losses because a surge is a translation of mass^11^. Others are undoubtedly due to poor DEM quality, for which we did not attempt any fixes because they covered a very small proportion (< 1 %) of the total glacier area. The remaining few examples are due to a fault in our workflow that cannot automatically account for situations where lateral moraine crests are lower in a transverse direction than the glacier surface; for example, in steeply convex ice falls or on piedmont lobes. Across the entirety of our dataset, the area covered by grid cells with positive elevation changes is 5.6% of the total glacier area and the magnitude of these positive values combined represents between 3 % and 4.4 % of the (lower and upper bounds of) volume loss that we compute.

Prescribing a convex profile to reconstructed ice surfaces is not trivial and subject to subjective decisions ^12^. In a sensitivity test [in their SI] Carrivick et al. ^9^ found typically < 5 % volume difference (but up to 30 % difference for some very small glaciers) after prescribing a convex surface compared to not, for individual glaciers. This is a very small sensitivity of the volume difference calculation, and virtually insignificant when the time frame of changes and hence rates of change are computed.

Uncertainty in area estimates will result from DEM resolution, LIA moraine identification and digitising precision. Our LIA area change estimates are subject to uncertainty depending on the hillshaded DEM (8 m) and optical image (< 1 m) resolution used for digitising moraine crests, and subjective choices of the most prominent inner moraine and of trimlines. Nevertheless, in the vast majority of cases the geomorphological evidence is distinct, whilst digitising errors for smaller glaciers will have the largest relative effect in area measurement accuracy^12^. For a typical Himalayan glacier of 2 km^2^, digitizing errors of one pixel would typically produce an area of ~ ± 2 % (depending on glacier shape), which is less than Nuimura et al.^12^ reported for their Himalaya glaciers but they were using 30 m raster imagery and included inexperienced operators and poorly-lit imagery. For the debris-covered ablation tongues, uncertainty might be as much as 10 %^12^.

Uncertainty in our volume estimates depend on the extraction of elevations from a DEM on the ablation area outlines. Due to the frequent holes in the HMA DEM coverage^13^, we were unable to use it for all glaciers. We therefore examined usage of the ALOS DEM^14^, which is seamless but of coarser 30 m resolution. We conducted an assessment of the effect of DEM source (timing of imagery used to construct DEM and resolution of DEM) on our volume estimates. Our findings, for the Langtang region that has good HMA DEM coverage, is that our LIA volume estimates either for individual glaciers or for entire regions do not depend on DEM source (Figure SI 1).

Our volume estimates do depend on the interpolation method used to derive a LIA glacier surface from points on moraine crests. For the interpolation, 4 different spatial interpolation techniques were considered: Natural Neighbour, Inverse Distance Weighting, Kriging, and Spline. Their accuracy in interpolating elevation was assessed by using a *K*-fold cross validation to evaluate their predictive methods. To do this, areas of known elevation (i.e. digitised points along the LIA moraine crest) were taken; each interpolation technique was iterated through 10 times with a random 10 % of the points removed for each iteration. The points removed (with the extracted interpolated elevation) were then assessed for their ‘closeness of fit’ with the actual elevation. The interpolated technique with the lowest root-mean-square-error (RMSE) and lowest standard deviation (STD) was determined to be the best method. Results of the analysis are shown within Table SI 1. As Carrivick et al.^8, 9^ also reported, Natural Neighbour was determined in this study to be the more realistic spatial interpolation routine to estimate LIA glacier surfaces from points on moraine crests. Natural Neighbour interpolation incurred the lowest RMSE of 10.49 m and the lowest STD of 13.18 m. Spline gave similar results with a RMSE of 10.66 m and a STD of 16.14 m but Spline does not preserve the original values of input points and thereby yields erroneous sinks and ridges. Carrivick et al.^9^ found that Kriging interpolation gave similar results to Natural Neighbour but was very computationally intensive, while additionally in our study Kriging incurred too high an error in its elevation prediction.

Rates of change do not depend so much on volume estimate, but rather are largely affected by the choice of a date for the LIA. Glaciers across the LIA reached their maximum LIA extent at different times^1^, but there is no trivial way to assign a date to individual glaciers, or even to individual regions. We therefore took the earliest (year 1300 CE) and latest (year 1600 CE) dates for the LIA across the Himalaya as proposed by Rowan^1^ and calculated rates of change for both scenarios. The shortest time, i.e. from year 1600, produces the most rapid centennial rate changes that are plausible, but these are still an order of magnitude slower than the rates reported for the last few decades by Brun et al.^15^, for example.

For the purposes of comparing our centennial-scale volume changes to contemporary changes reported in the literature, and for evaluating the relative importance of Himalayan glaciers to global sea level contributions, we converted our volume changes into mass changes. For this calculation, we used an ice density of 850 kg/m^3^ from Huss^16^. That density assumes, for simplicity, that all volume lost was ice, even though a small proportion could have been firn, and it complies with our aim for a conservative estimate of ice volume loss. The mass of ice was converted to a sea level equivalent (SLE) using an ocean area of 3.62 x 10^8^ km^2^ from Hock et al.^17^.


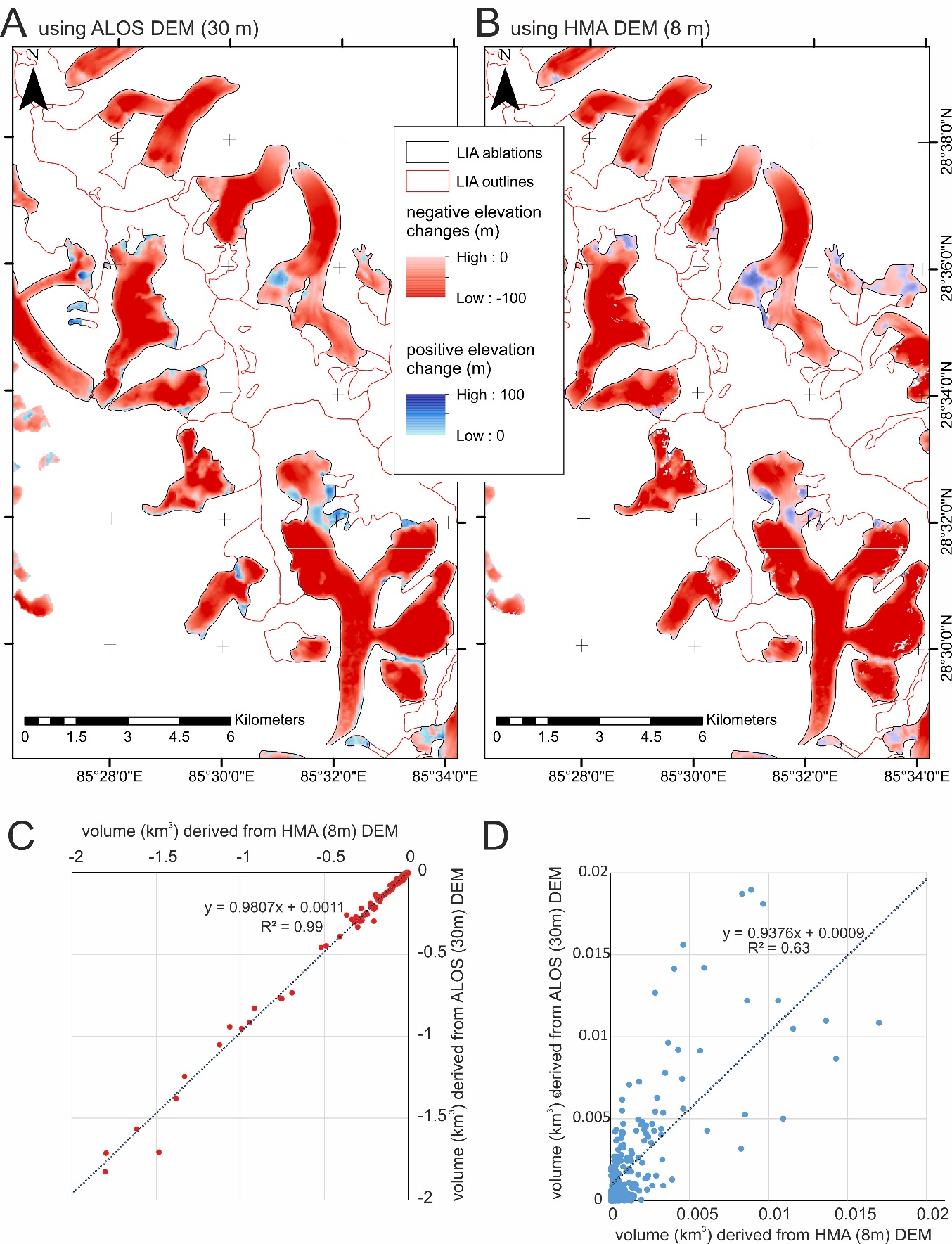


**Figure SI 1**. Effect of DEM source on surface lowering (maps) and volume changes per glacier (graphs) for the Langtang region. A) The 30 m ALOS DEM, B) the 8 m HMA DEM, C) negative elevation and D) positive elevation, changes compared between the two DEMs. The dataset analysis and preparation of parts A and B of this figure were made using ESRI ArcGIS software (v. 10.6).

|  | NN | | IDW | | Kriging | | Spline | |
| --- | --- | --- | --- | --- | --- | --- | --- | --- |
| Iteration | RSME | STD | RMSE | STD | RSME | STD | RSME | STD |
| 1 | 10.39 | 13.73 | 16.75 | 20.91 | 15.63 | 20.83 | 11.07 | 16.02 |
| 2 | 10.22 | 12.5 | 16.39 | 21.58 | 15.47 | 19.25 | 10.47 | 17.87 |
| 3 | 10.18 | 12.61 | 16.66 | 21.55 | 16.7 | 21.08 | 10.74 | 16.96 |
| 4 | 10.77 | 13.98 | 17.07 | 21.76 | 16.48 | 19.73 | 9.98 | 13.16 |
| 5 | 10.65 | 13.05 | 15.38 | 18.98 | 15.9 | 19.22 | 10.64 | 18.41 |
| 6 | 10.62 | 13.49 | 16.78 | 21.42 | 16.52 | 20.62 | 11.2 | 15.36 |
| 7 | 10.35 | 12.6 | 16.84 | 20.04 | 16.25 | 19.71 | 10.59 | 14.62 |
| 8 | 10.68 | 13.29 | 17.48 | 22.08 | 16.88 | 21.04 | 11.17 | 20.13 |
| 9 | 10.59 | 12.84 | 15.68 | 20.49 | 20.37 | 24.31 | 10.62 | 16.04 |
| 10 | 10.47 | 13.68 | 16.85 | 21.3 | 16.17 | 19.06 | 10.08 | 12.87 |
| Mean | 10.49 | 13.18 | 16.59 | 21.01 | 16.63 | 20.49 | 10.66 | 16.14 |

**Table SI 1**. K-Fold Cross Validation results for spatial interpolation of LIA glacier surfaces from point elevations on moraine ridge crests.

### Determination of glacier type and terminus environment

Studies have identified that the glacial terminus conditions can affect the rate of glacial mass loss^18^. In this study, glaciers were assigned to one of four mutually exclusive classes depending on their surface characteristics (debris-covered vs clean-ice) and their terminus environment (lacustrine or on land). Hereon-in we refer to those four classes accordingly: debris-lake (1%), debris-land (7%), clean-lake (4%) and clean-land (88%).

Lake-terminating glaciers were identified via the inventory of High Mountain Asia glacial lakes from Wang et al.^19^ for 1990 and 2018, which was derived from 30 m Landsat imagery; we used the 2018 inventory. Wang et al.^19^ used a normalized difference water index (NDWI) to identify water bodies, while any water bodies within a 10 km buffer of a glacier terminus were determined as a glacial lake. In this study, a ‘selection by location’ was performed to select all glaciers that were within a 30 m buffer of a glacial lake.

Glaciers were identified with ‘debris-cover’ via a dataset from Kraaijenbrink et al.^20^, which includes debris-cover for glaciers across the Hindu-Kush-Himalaya region. This dataset was created from Landsat 8 imagery and by selecting pixels below a normalized difference snow index (NDSI) value of < 0.25 and a slope threshold of < 24° to a debris cover pixel. In this study, glacier outlines were then assigned a percentage cover from this debris-cover raster layer and any glacier with > 30 % debris cover was categorised as ‘debris-covered’.

Glaciers were identified to be of both debris-cover and lake-terminating condition where the results from the above two categorisations coincided spatially. The remaining glaciers in our study were by default ‘land-terminating’ and ‘clean ice’.

In our categorisation of glacier type and terminus environment, any single glacier is assigned to one group only. In reality, glaciers can exhibit several of the properties simultaneously and to varying degrees. However, we draw attention to the fact that Brun et al.^18^ have shown that different thresholds used to categorise glaciers into their respective terminating conditions have limited influence on the final results. Furthermore, it must be acknowledged that our categorisations are based on modern conditions. Both debris-cover and proglacial lakes evolve through time and we cannot say anything about this evolution since the LIA.

We predominantly analysed glacier changes by morpho-climatic zone following those of Brun et al.^15^. It should be noted that with these zones the glaciers of Sikkim are part of the category East Nepal and that all glaciers from Uttarakhand are integrated in the category Spiti-Lahaul. Glacier volume changes between the LIA to present were -148 km^3^ for Spiti-Lahaul, -107 km^3^ for West Nepal, -170 km^3^ for East Nepal and -63 km^3^ for Bhutan. By major river catchment/drainage basin this glacier volume change is -160 km^3^ for the Indus, -234 km^3^ for the Ganges and -93 km^3^ for the Brahmaputra. Our analysis of glacier changes for north versus south of the main divide and for major river catchment/drainage basin is summarised in Figure SI 2.

***Statistical difference of mass loss between groups***

GLMs (Generalised Linear Models) were constructed in R3.6.1^21^ to assess if there was a statistically significant difference between differing groups. The GLM approach allowed us to account for non-normally distributed mass balance data. Mass-balance values (which are a negative m w.e.) were inverted prior to analysis. Zero-inflated Poisson distribution models were specified using the pscl package (<https://www.rdocumentation.org/packages/pscl>) to account for zero observations, which arose due to some small fragments of ablation areas produced by the automatic extraction of ablations areas; i.e. where multiple cirques feed into a single ablation tongue some small lowermost parts of some cirques were classified as ablation areas but were disconnected from the main tongue. We observed difference in mass balance between groups, namely; (i) between region (p=0.024; McFadden pR^2^=0.06, (ii) north versus south of the main divide (p=0.0471; pR^2^=0.06), and (iii) between glacier terminus and surface type (p=4.5e^-08^; pR^2^=0.03). We also ran nested models: region/glacier type in (p=3.85e^-07^; pR^2^=0.07), north versus south/glacier type (p=1.65e^-05^; pR^2^=0.10), and region/north versus south/glacier type (p= 3.17e^-05^ ; pR^2^=0.06) (Table SI2). The large proportion of unexplained variance across all models is likely a reflection of local topographic factors which could not be quantified within our remote sensing framework.


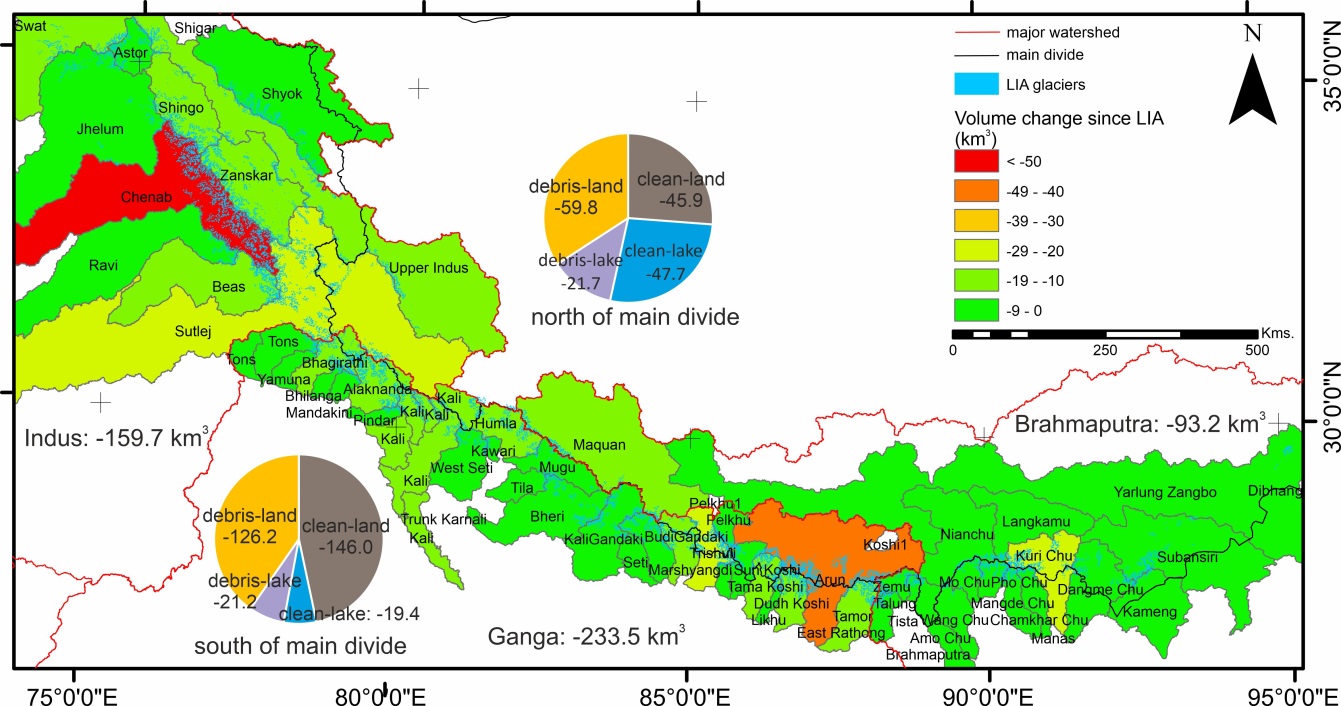


**Figure SI 2.** Total volume change of glaciers since the LIA discriminated by drainage basin and by glacier terminus type north/south of the main divide. The dataset analysis and preparation of this figure was made using ESRI ArcGIS software (v. 10.6).

|  |  |  | **1300** | **1600** | **1300** | **1600** |
| --- | --- | --- | --- | --- | --- | --- |
| **Region** | **Terminus type** | **n** | **median m w.e/yr** | | **IQR m w.e./yr** | |
| Bhutan |  | 1356 | -0.012 | -0.022 | 0.015 | 0.027 |
| East Nepal |  | 2380 | -0.013 | -0.023 | 0.016 | 0.029 |
| West Nepal |  | 2902 | -0.011 | -0.019 | 0.012 | 0.022 |
| Spiti Lahaul |  | 8160 | -0.009 | -0.016 | 0.010 | 0.018 |
| North (all) |  | 3601 | -0.011 | -0.020 | 0.015 | 0.025 |
| South (all) |  | 11197 | -0.010 | -0.017 | 0.011 | 0.020 |
| Bhutan North | clean-land | 778 | -0.010 | -0.017 | 0.013 | 0.023 |
|  | clean-lake | 100 | -0.025 | -0.043 | 0.020 | 0.034 |
|  | debris-lake | 6 | -0.022 | -0.039 | 0.038 | 0.067 |
|  | debris-land | 55 | -0.020 | -0.035 | 0.014 | 0.025 |
| Bhutan South | clean-land | 296 | -0.010 | -0.018 | 0.012 | 0.020 |
|  | clean-lake | 83 | -0.023 | -0.039 | 0.013 | 0.023 |
|  | debris-lake | 14 | -0.030 | -0.052 | 0.025 | 0.044 |
|  | debris-land | 24 | -0.025 | -0.043 | 0.012 | 0.022 |
| East Nepal North | clean-land | 924 | -0.010 | -0.018 | 0.015 | 0.025 |
|  | clean-lake | 147 | -0.026 | -0.045 | 0.023 | 0.040 |
|  | debris-lake | 20 | -0.037 | -0.064 | 0.027 | 0.047 |
|  | debris-land | 165 | -0.022 | -0.038 | 0.025 | 0.043 |
| East Nepal South | clean-land | 839 | -0.011 | -0.019 | 0.014 | 0.024 |
|  | clean-lake | 90 | -0.026 | -0.045 | 0.015 | 0.026 |
|  | debris-lake | 26 | -0.030 | -0.052 | 0.020 | 0.036 |
|  | debris-land | 169 | -0.018 | -0.031 | 0.014 | 0.024 |
| West Nepal North | clean-land | 598 | -0.010 | -0.018 | 0.012 | 0.021 |
|  | clean-lake | 46 | -0.021 | -0.036 | 0.015 | 0.026 |
|  | debris-lake | 8 | -0.005 | -0.008 | 0.018 | 0.032 |
|  | debris-land | 41 | -0.015 | -0.027 | 0.009 | 0.016 |
| West Nepal South | clean-land | 1902 | -0.010 | -0.017 | 0.012 | 0.021 |
|  | clean-lake | 49 | -0.015 | -0.026 | 0.009 | 0.016 |
|  | debris-lake | 6 | -0.020 | -0.036 | 0.011 | 0.020 |
|  | debris-land | 252 | -0.016 | -0.028 | 0.012 | 0.022 |
| Spiti Lahaul North | clean-land | 677 | -0.009 | -0.016 | 0.011 | 0.019 |
|  | clean-lake | 14 | -0.017 | -0.030 | 0.007 | 0.013 |
|  | debris-lake | 0 | - | - | - | - |
|  | debris-land | 22 | -0.013 | -0.023 | 0.009 | 0.016 |
| Spiti Lahaul South | clean-land | 7072 | -0.009 | -0.016 | 0.010 | 0.018 |
|  | clean-lake | 64 | -0.016 | -0.028 | 0.010 | 0.017 |
|  | debris-lake | 13 | -0.020 | -0.035 | 0.015 | 0.027 |
|  | debris-land | 298 | -0.015 | -0.025 | 0.012 | 0.020 |

**Table SI 2.** Mass balance from the LIA between data groups.

|  | Gardelle et al. (2013) | Kääb et al. (2015) | Brun et al. (2017) | Maurer et al. (2019) | | This study | |
| --- | --- | --- | --- | --- | --- | --- | --- |
| Time period | 2000-2010 | 2003-2008 | 2000-2016 | 1975-2000 | 2000-2016 | 1300-2020 | 1600-2020 |
| Spiti Lahaul | -0.45±0.14 | -0.42±0.26 | -0.37±0.09 | -0.15±0.14 | -0.45±0.15 | -0.010 | -0.017 |
| West Nepal | -0.32±0.14 | -0.37±0.15 | -0.34±0.09 | -0.28±0.13 | -0.38±0.14 | -0.012 | -0.021 |
| East Nepal | -0.26±0.14 | -0.31±0.14 | -0.33±0.20 | -0.22±0.12 | -0.41±0.12 | -0.016 | -0.028 |
| Bhutan | -0.22±0.14 | -0.76±0.20 | -0.42±0.20 | -0.25±0.12 | -0.51±0.15 | -0.014 | -0.025 |
| Mean | -0.35±0.08 | -0.42±0.12 | -0.36±0.07 | -0.21±0.07 | -0.43±0.08 | -0.013 | -0.023 |

**Table SI 3.** Comparisons with prior studies for mass balance (m w.e./yr) within the Himalaya

**References**

1. Rowan AV. The ‘Little Ice Age’ in the Himalaya: A review of glacier advance driven by Northern Hemisphere temperature change. *The Holocene* 2017, **27**(2)**:** 292-308.

2. Holzhauser H, Magny M, Zumbuühl HJ. Glacier and lake-level variations in west-central Europe over the last 3500 years. *The Holocene* 2005, **15**(6)**:** 789-801.

3. Deota B, S., Trivedi Y, N., Kulkarni A, V., Mankad M, D. Reconstructing Glacial History of Jorya Garang Glacier from Little Ice Age to Present. *Journal of Geosciences Research* 2018, **3**(2)**:** 163-170.

4. Peng X, Chen Y, Li Y, Liu B, Liu Q, Yang W*, et al.* Late Holocene glacier fluctuations in the Bhutanese Himalaya. *Global and Planetary Change* 2020, **187:** 103137.

5. Pellitero R, Rea BR, Spagnolo M, Bakke J, Hughes P, Ivy-Ochs S*, et al.* A GIS tool for automatic calculation of glacier equilibrium-line altitudes. *Computers & Geosciences* 2015, **82:** 55-62.

6. Rea BR. Defining modern day Area-Altitude Balance Ratios (AABRs) and their use in glacier-climate reconstructions. *Quaternary Science Reviews* 2009, **28**(3)**:** 237-248.

7. RGI Consortium. Randolph Glacier Inventory – A Dataset of Global Glacier Outlines: Version 6.0: Technical Report, Global Land Ice Measurements from Space. Colorado, USA; 2017.

8. Carrivick JL, James WHM, Grimes M, Sutherland JL, Lorrey AM. Ice thickness and volume changes across the Southern Alps, New Zealand, from the little ice age to present. *Scientific Reports* 2020, **10**(1)**:** 13392.

9. Carrivick JL, Boston CM, King O, James WHM, Quincey DJ, Smith MW*, et al.* Accelerated Volume Loss in Glacier Ablation Zones of NE Greenland, Little Ice Age to Present. *Geophysical Research Letters* 2019, **46**(3)**:** 1476-1484.

10. Carrivick JL, Brewer TR. Improving local estimations and regional trends of glacier equilibrium line altitudes. *Geografiska Annaler: Series A, Physical Geography* 2004, **86**(1)**:** 67-79.

11. Benn DI, Fowler AC, Hewitt I, Sevestre H. A general theory of glacier surges. *Journal of Glaciology* 2019, **65**(253)**:** 701-716.

12. Nuimura T, Sakai A, Taniguchi K, Nagai H, Lamsal D, Tsutaki S*, et al.* The GAMDAM glacier inventory: a quality-controlled inventory of Asian glaciers. *The Cryosphere* 2015, **9**(3)**:** 849-864.

13. Shean D. High Mountain Asia 8-meter DEM Mosaics Derived from Optical Imagery, Version 1. Boulder, Colorado USA: NASA National Snow and Ice Data Center Distributed Active Archive Center; 2017.

14. Tadono T, Ishida H, Oda F, Naito S, Minakawa K, Iwamoto H. Precise Global DEM Generation by ALOS PRISM. *ISPRS Ann Photogramm Remote Sens Spatial Inf Sci* 2014, **II-4:** 71-76.

15. Brun F, Berthier E, Wagnon P, Kääb A, Treichler D. A spatially resolved estimate of High Mountain Asia glacier mass balances from 2000 to 2016. *Nature Geoscience* 2017, **10**(9)**:** 668-673.

16. Huss M. Density assumptions for converting geodetic glacier volume change to mass change. *The Cryosphere* 2013, **7**(3)**:** 877-887.

17. Hock R, de Woul M, Radić V, Dyurgerov M. Mountain glaciers and ice caps around Antarctica make a large sea-level rise contribution. *Geophysical Research Letters* 2009, **36**(7).

18. Brun F, Wagnon P, Berthier E, Jomelli V, Maharjan SB, Shrestha F*, et al.* Heterogeneous Influence of Glacier Morphology on the Mass Balance Variability in High Mountain Asia. *Journal of Geophysical Research: Earth Surface* 2019, **124**(6)**:** 1331-1345.

19. Wang X, Guo X, Yang C, Liu Q, Wei J, Zhang Y*, et al.* Glacial lake inventory of high-mountain Asia in 1990 and 2018 derived from Landsat images. *Earth Syst Sci Data* 2020, **12**(3)**:** 2169-2182.

20. Kraaijenbrink PDA, Bierkens MFP, Lutz AF, Immerzeel WW. Impact of a global temperature rise of 1.5 degrees Celsius on Asia’s glaciers. *Nature* 2017, **549**(7671)**:** 257-260.

21. R Core Team. R: A language and environment for statistical computing. Vienna, Austria: R Foundation for Statistical Computing; 2018.
